# Light-stimulus intensity modulates startle reflex habituation in larval zebrafish

**DOI:** 10.1038/s41598-021-00535-9

**Published:** 2021-11-17

**Authors:** Carolina Beppi, Giorgio Beringer, Dominik Straumann, Stefan Yu Bögli

**Affiliations:** 1 Neuroscience Center Zurich, University of Zurich and ETH Zurich, 8091 Zurich, Switzerland; 2 Department of Neurology, University Hospital Zurich and University of Zurich, Frauenklinikstrasse 26, 8091 Zurich, Switzerland; 3 Clinical Neuroscience Center, University Hospital Zurich and University of Zurich, 8091 Zurich, Switzerland; 4 Swiss Concussion Center, Schulthess Clinic, 8008 Zurich, Switzerland

**Keywords:** Neurophysiology, Reflexes, Behavioural methods

## Abstract

The startle reflex in larval zebrafish describes a C-bend of the body occurring in response to sudden, unexpected, stimuli of different sensory modalities. Alterations in the startle reflex habituation (SRH) have been reported in various human and animal models of neurological and psychiatric conditions and are hence considered an important behavioural marker of neurophysiological function. The amplitude, offset and decay constant of the auditory SRH in larval zebrafish have recently been characterised, revealing that the measures are affected by variation in vibratory frequency, intensity, and interstimulus-interval. Currently, no study provides a model-based analysis of the effect of physical properties of light stimuli on the visual SRH. This study assessed the effect of incremental light-stimulus intensity on the SRH of larval zebrafish through a repeated-measures design. Their total locomotor responses were normalised for the time factor, based on the behaviour of a (non-stimulated) control group. A linear regression indicated that light intensity positively predicts locomotor responses due to larger SRH decay constants and offsets. The conclusions of this study provide important insights as to the effect of light properties on the SRH in larval zebrafish. Our methodology and findings constitute a relevant reference framework for further investigation in translational neurophysiological research.

## Introduction

The startle reflex in larval zebrafish is a widely studied evolutionary-preserved escape response to sudden/unexpected—potentially threatening—stimuli^1^, occurring in the form of C-shaped bending of the body, known as ‘C-starts’^2–5^. It can be induced by different sensory modalities, including the visual, auditory, and somatosensory^6–9^ and it is largely mediated by Mauthner interneurons^6–8^, although not exclusively^10^. Brief light flashes can cause C-starts with average latency of ~ 183 ms^4^, and their frequency (i.e., proportion/number of motor responses per stimulus) is proportional to contrast to baseline lighting levels^4^. Startle responses have also been probed using looming visual stimuli^11^. Dark light flashes can likewise trigger reflex turns (*O-turns*), although of prolonged latency (mean =  ~ 408 ms) and larger bend angle^4^ compared to classic visually evoked C-starts. Endogenous modulating factors of the startle reflex include arousal and stress levels^12,13^, which can be increased by sensitisation or fear-potentiation^14^ and decreased by pre-pulse inhibition^15,16^^.^

Startle reflex habituation (SRH) constitutes a basic, non-associative form of learning whereby repeated or continued exposure to an unexpected stimulus results in reduced motor responsivity^9,17,18^. It has been studied in several animal species^19–23^ and humans^24^. Larval zebrafish SRH is reliably inducible, although not stereotypic (i.e., liable to variability and individual differences^12,13,25^. Habituation can be rapid and very short-lasting (≤ 15 min), short-lasting (hours), or long-lasting (days), depending on how prolonged the exposure to stimulation is^10^. The SRH is comprised of peripheral and central components. The first is a sensory adaptation that involves the saturation of transducing cells after repeated exposure to the same sensory stimulus^26–30^. The central component of SRH is a habituation *sensu stricto* that reduces the motor reaction to sudden sensory stimuli, if regarded as non-threatening/relevant, despite being still processed at primary cortical level^31–34^. Both peripheral and central components of larval zebrafish SRH are induced by high-frequency stimuli^35^, while the peripheral component, i.e., sensory adaptation, may fail when longer inter-stimuli intervals ((ISIs), ≥ of 3 s) are utilised^35^. Alterations in startle reflex amplitude and SRH characterise several neuropsychiatric conditions^36–41^ and hence constitute important markers of neurophysiological function used in translational research (see Kalueff et al.^42^; Stewart et al.^43^).

Evidence suggests that startle response frequency of larval zebrafish to single white-light-flash stimuli depends on the magnitude-change in lighting^4^, although Beppi et al. in a recent study using the same stimuli found no such effect^35^. However, Burgess and Granato^4^ quantified the escape responses distinguishing ‘scoot’ (< 30–40°) and ‘R-turn’ (> 30–40°) initiations, observing that only the latter increase in frequency with stronger light intensities. In contrast, Beppi et al.^35^ did not distinguish scoot and R-turn initiations in their SRH quantifications and hence their global effect size might have been deflated. Moreover, Burgess and Granato^4^ pre-adapted the fish at ~ 135 Lux and tested them with light stimuli of ~ 1366 Lux (i.e., 90% step increase in lighting), while Beppi et al.^35^ pre-adapted the fish at ~ 330 Lux and tested them with a step-increase in lighting ranging from 35 to 64%. Therefore, the authors suggested reapplying their experimental protocol with stronger light intensities to test whether this would result in findings consistent with the literature.

### Aims and hypotheses

This study aimed at following-up the study of Beppi et al.^35^, reproducing their visual SRH study protocol using stronger lighting conditions (from 0.5 to 16 kLux), to test the effect of different luminance levels on the SRH of larval zebrafish of the same age. We hypothesised that the stimulated group would show a significantly larger mean total (of all 20 stimuli) distance travelled (TDT_20_) relative to the control group, increasing along with higher lux levels. We further hypothesised that such increase in TDT_20_ would be underlain by changes of exponential SRH parameters (i.e., amplitude, decay constant or offset).

## Results

**Figure 1:**
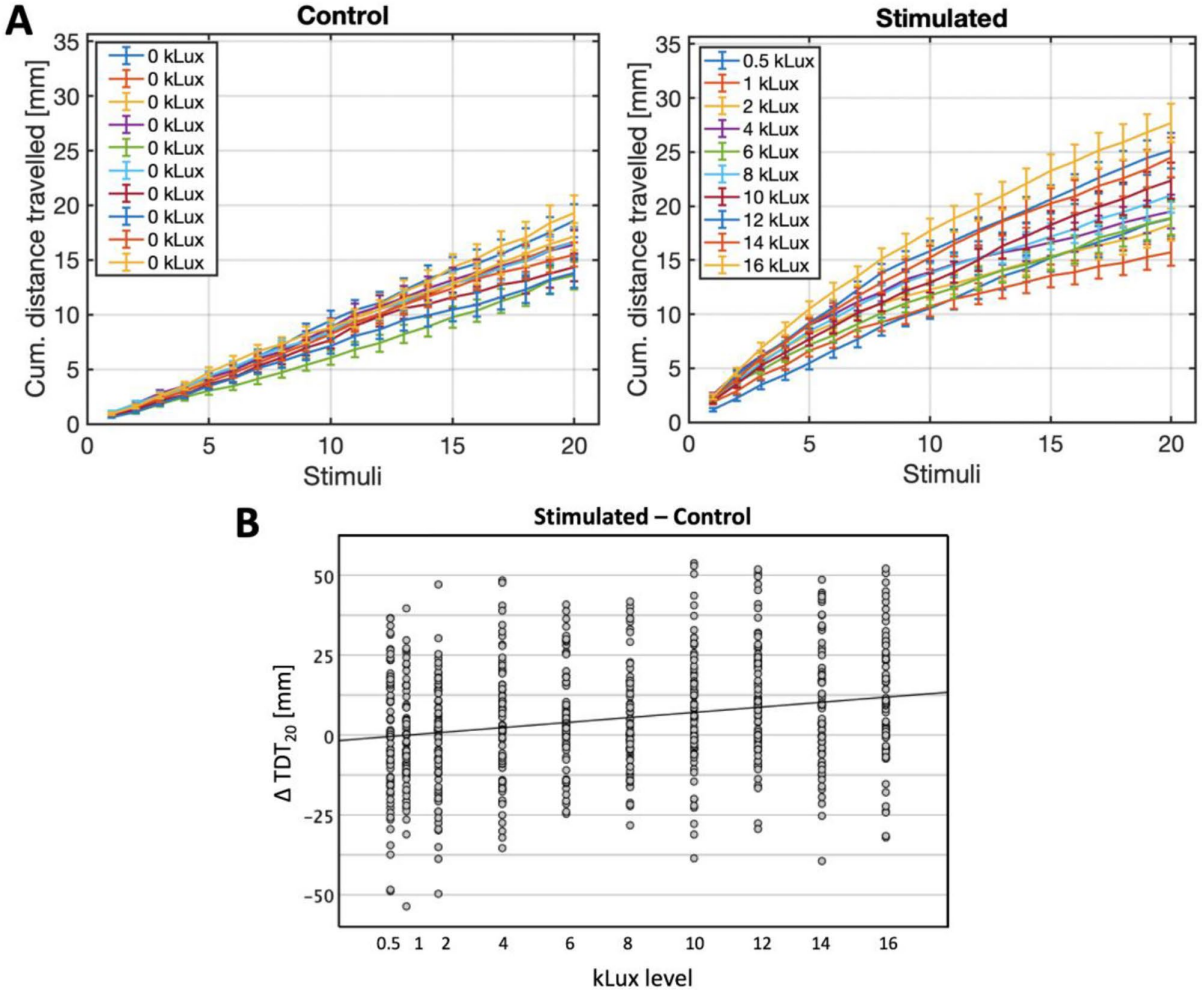
(**A**) Mean distance travelled (± 1 SE) cumulatively over stimuli, for the stimulated group (right panel) and the respective—with same line colour—control group (left panel), at each kLux level. (**B**) Mean change in TDT_20_ (± 1 SE) of the stimulated group relative to the control, at each kLux level (each dot represents a single larva). The predictive model was: Δ TDT_20_ = − 0.84 + 0.794*(kLux level).

**Figure 2:**
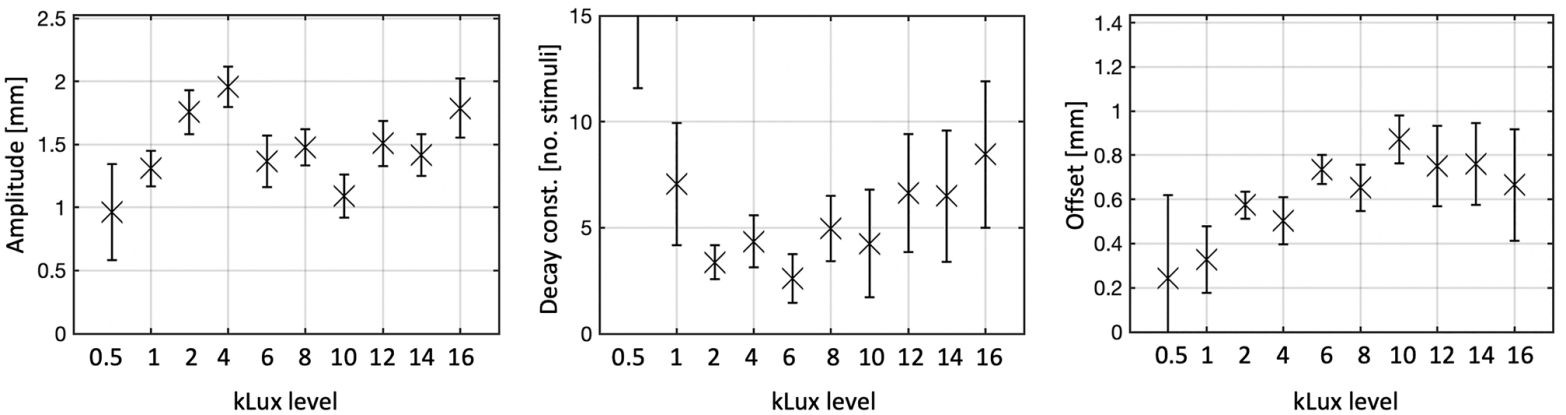
Boxplots for the mean (cross; ± 1 SE) amplitude, decay constant and offset of 500 bootstraps, for both groups, at each kLux level.

A simple visual SRH task was applied repeatedly over 10 runs during which the larvae were stimulated at incrementing lux levels from 0.5 to 16 kLux (1 run = 20 stimuli). Plots of the groups’ mean distance travelled in each lux-level condition, cumulatively over successive stimuli, are shown in Fig. 1A, while mean TDT_20_ values for each group, at each lux level, are reported in Table 1 (supplementary information). The TDT_20_ of the stimulated group was normalised for the effect of time, subtracting the free locomotor distance travelled by a control group, which was tested concomitantly to the experimental group at all the runs in the absence of additional light stimulation. The TDT_20_ distribution of both groups at all test-times approximately met the assumptions for single linear regression analysis, which was therefore performed to predict the change in TDT_20_ (relative to control) as an effect of light intensity (lux). The lux level significantly explained 5.1% of variance in mean TDT_20_, with F(1,718) = 38.326, p < 0.001. A one-unit increase in lux causes a control-relative positive change in mean TDT_20_ of 0.794 (95% CI [0.542, 1.046]). The predictive model is shown in Fig. 1B. The population-level (bootstrapped) amplitude, decay constant and offset are illustrated in Fig. 2.

## Discussion

The startle reflex in larval zebrafish is an evolutionary preserved escape response to potential threat, occurring in different sensory modalities^1–11^. The SRH is a basic non-associative form of learning, evidenced by a reduction in motor responsivity after repeated or continued exposure to a startling stimulus^9,17,18^. The SRH in larval zebrafish, although non-stereotypic, is reliably inducible^12,13,25^. Recently, Beppi et al.^35^ have proposed a first-order exponential model description of SRH in larval zebrafish, showing that its three main descriptive measures—amplitude, decay constant and offset—are sensitive to variation in vibratory frequency and intensity as well as ISI duration. However, no variability in such SRH measures was found as an effect of light stimulation within 0.4 kLux.

This study aimed at assessing the effect of light-stimulus intensities ≥ 0.5 kLux (with a step-increase in contrast from baseline lighting > 64%) on SRH of 72 larval zebrafish, based on the model-based analytical approach of Beppi et al.^35^^.^ A simple visual SRH task was conducted repeatedly in 10 runs stimulating at incremental kLux levels. We predicted an increase in TDT_20_ and associated changes in SRH parameters—amplitude, decay constant or offset—along with higher stimulatory lux levels. In line with our first hypothesis, the linear regression analysis revealed that light intensity was a significant positive predictor of locomotor responses. The stimulated group showed a modest—but highly significant—increase in TDT_20_ relative to the control group, along with higher light-stimuli intensity. The mean TDT_20_ value of the fish stimulated at the lowest 3 kLux levels was comparable to those of the control group, while an increase in TDT_20_ relative to control started to be visible at 4 kLux (~ 19%), increasing up to > 80% in the highest lux levels. Noticeably, when stimulated at 0.5 kLux, the fish showed a markedly lower amplitude of the exponential SRH fit compared to when tested at higher lux levels. This suggests that 0.5 kLux might not be sufficient for inducing a reliable SRH. This is further confirmed by unrealistically large decay constant for the 0.5 and 1 kLux levels, relative to the higher ones. It is hence recommendable for future SRH experiments in larval zebrafish (performed at environmental levels) to stimulate with light flashes of at least 2 kLux.

The fitting of the locomotor data with a single exponential—with parameters amplitude, decay constant and offset—highlights a consistent trend of enlargement of the decay constants starting from 2000 towards higher lux levels. This effect is concomitant to an increase in offset along with higher lux levels (although approaching a ceiling at 10 kLux) and a general reduction (although with high variability) in amplitude in the kLux levels beyond 4. The increasing of both offset and decay constant might be product of the retinal *bleaching effect*—generally defined as a reduction in light sensitivity of the photoreceptors (i.e., adaptation) after extensive rhodopsin photoconversion^44,45^. Specifically to our case, if the fish are stimulated with high kLux levels (i.e., 10 or higher), the retinal representation of the image might last longer (stronger sensory memory), presumably beyond the ISI duration, as if the fish were exposed to “permanent light”. This would cause an increasing (perceived) magnitude of subsequent stimuli—as a putative linear or asymptotic trend—in turns leading to a fictitious increase of offset and decay constant of the single exponential fit, as we could observe in our plots.

**Figure 3:**
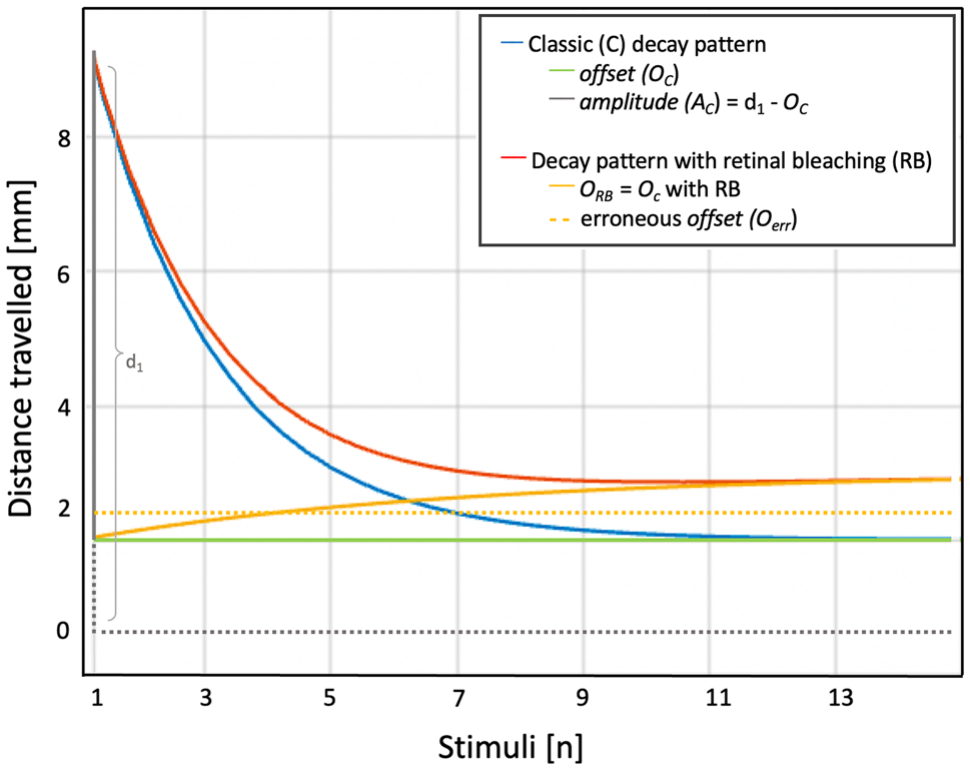
Depiction of the input of *retinal bleaching* (RB) to the startle habituation, using simulated data. In the classic decay model (blue) described by Beppi et al.^35^, the amplitude (*A*_*C*_) corresponds to the magnitude of the locomotor response to the first stimulus (d_1_) minus the offset (*O*_*C*_), namely the steady-state responsivity (constant). The decay pattern with RB accounts for the effect of retinal bleaching with decay to an asymptotically increasing offset (*O*_*RB*_). Habituation is a high-pass filter [*A*_*C*_ × exp(Dc × *n*)], where Dc is the decay constant and *n* the stimulus number minus 1. The bleaching effect is instead a low-pass filter [*A*_*RB*_ × exp(–*n* / Rc)] where Rc is the rise constant and is larger than Dc (in this example Dc = 1 and Rc = 3), while *A*_*RB*_ (= d_1_ – *O*_*RB*_) is considerably smaller than *A*_*C*_. The habituation with bleaching effect can hence be described as [*A*_*C*_ × exp(Dc × *n*) + *A*_*RB*_ × exp(–*n* / Rc) + *O*_*C*_]. When trying to fit a classic first-order exponential into such a decay pattern with retinal bleaching, a “fictitiously” high average (i.e., constant) offset (*O*_*err*_) and a “fictitiously” smaller amplitude (*A*_*err*_ = d_1_ – *O*_*err*_) would be obtained, as we can observe in Figure 2 (see offsets and amplitudes in the highest lux-levels).

Our data did not allow the fitting of more complex functions than first-order exponentials. However, one could consider adding a low-pass filter on the fitted data to model the retinal receptor property causing subsequent stimuli to be perceived with increasing magnitude (see Fig. 3 for a simulation of a decay pattern with retinal bleaching). The application of electro-retinogram protocols (e.g.^46,47^) would be valuable to monitor the visual function during the SRH task and for testing the validity of these assumptions. We therefore suggest its complementary use in future light-induced SRH studies in larval zebrafish.

## Conclusions

Our results show that the visual SRH incurs consistent changes as a function of systematic increases in light-stimulus intensity. As the stimulation intensity increases, so does the TDT_20_. The underlying habituation metrics show however distinct outcome patterns. While the SRH decay constant—starting from 2 kLux stimuli and stronger—increases linearly along with higher intensities, the offset increases reaching a ceiling at ~ 10 kLux. The amplitude was instead the habituation parameter showing the highest variability across the kLux levels. Stimulation intensities of about 2–4 kLux where in general the most preferable, showing a relatively higher amplitude, lower offset and lower decay constant—which are all indicative of effective habituation.

In addition, the results confirm and extend past literature findings, evidencing that higher light intensities produce increased locomotor responses, not only in terms of frequency^4^, but also in terms of length of distance travelled. The findings also extend our previous model-based analysis of light-induced SRH in larval zebrafish^35^, validating its methodology and showing that—if sufficiently intense—light is a significant predictor of TDT_20_, and can differentially influence exponential SRH parameters of amplitude, decay constant and offset. This study has provided novel insights as to the influence of light properties on the SHR profile over time in larval zebrafish.

Changes in SRH characterise several neurological and psychiatric conditions, such as attention-deficit hyperactivity disorder^36^, schizophrenia^37^, brain trauma^38^, generalised anxiety^39^ depressive^40^ and obsessive–compulsive disorders^41^. This study can hence constitute a framework of reference for translational research, providing easily-reproducible normative findings of SRH in larval zebrafish which can be used for quantifying the extent of CNS dysfunction in zebrafish models of neuropsychiatric disorders.

## Methods

### Ethical approval

The study was performed in accordance with the animal welfare guidelines of the Swiss Federal Veterinary Office, and all animal experiments were approved by the ethics committee of the Veterinary Office of the Canton of Zurich (ZH190/2020, 32971). The study and methods were conducted also in compliance with the ARRIVE guidelines of animal research.

### Fish maintenance, mating and egg production

Wild Indian Karyotype (WIK) *Danio rerio* adult zebrafish were bred and maintained in accordance with standard protocols^48^. Their eggs were raised in transparent breeding tanks of 1.7 L water, incubated at 28 °C, under a 14:10 light–dark cycle. Upon reaching 3–4 DPF, they were moved into 35 × 12 mm cell-culture plates filled with E3 medium (solution in mM: 5 NaCl, 0.17 KCl, 0.33 CaCl_2_, and 0.33 MgSO_4_; Sigma-Aldrich Corp., St. Louis, MO, USA) and kept in the same location until the test day.

### Material

The Viewpoint Zebrabox (ViewPoint Life Sciences, Lyon, France) behavioural recording system and software was used to track the locomotor responses of the animals in response to light stimulation. The light source consisted of a rectangular LED emitting white whole-field light flashes from beneath the 24-well plates.

### Experimental procedure

On the test day, zebrafish larvae of 92–96 h past fertilization (N = 144) were moved from the cell-culture plates and distributed in six 24-well plates filled with fresh E3 medium—half of which were randomly assigned to the control condition (three 24-well plates, N = 72), and the remaining half to the experimental condition (three 24-well plates, N = 72). The samples’ size provided sufficient statistical power. After an acclimatisation time of 90 min in the new plates, the testings took place. The experimental group performed the behavioural task at a given kLux level, while the control group was always tested at baseline (environmental) lighting level (~ 0.33 kLux) without additional light stimulation, for the same time duration of the experimental group’s task. Each of the six plates was tested consecutively, in its respective condition (control or experimental). The plates were tested with 1–2 min of time-lag between one another. All the groups performed the task ten times (10 runs). Each run consisted of a series of 20 identical white light flashes lasting 500 ms of a given kLux level with instantaneous onset/offset (step pattern). Each stimulus was interleaved by ISIs of 1000 ms, where the illuminance remained at baseline level. At each successive run, the kLux level of the experimental group incremented (0.5, 1, 2, 4, 6, 8, 10, 12, 14, 16), while the control group always performed the no-stimulation protocol. The first run began at ~ 11 a.m. Each of the following runs were interleaved by 10–12 min from the previous. During all testings, the light exposure of the fish in the ZebraBox was kept at environmental levels for the purpose of maintaining their normal circadian rhythm and avoid affecting their arousal state, which could result in unwanted motility changes.

### Quantitative modelling

Startle responses were quantified based on Beppi et al.^35^ in terms of locomotor distance travelled [mm] within the time frame of each stimulus (500 ms), for each fish of both groups. Movements that occurred during each ISI (1000 ms) were not quantified. The total distance travelled at all 20 stimuli (TDT_20_) was then computed for each fish of both groups. Lastly, the control-relative change in TDT_20_—e.g., mean of the control group subtracted from that of the stimulated group, for each respective kLux level—was linearly regressed to model its relationship with the illuminance level. Data bootstrapping (N = 500, in-built MATLAB function: bootstrp) was applied—at the individual fish level—on the distance travelled over the stimuli by the experimental group (N = 72), at each test-time (i.e., kLux level). This was done to obtain a “population-level” description of behaviour. Based on the SRH model described by Beppi et al.^35^, a first-order exponential (in-built MATLAB function: lsqcurvefit) was fitted into each individual bootstrap (N = 500), to extract individual *offset*, *amplitude,* and *decay constant*—the three main descriptive measures of startle habituation (for a definition of the measures, please refer to Beppi et al.^35^). These measures where then averaged, to obtain group-level mean measures.

### Statistical analyses

Statistical analyses were conducted using MATLAB R2020b (The MathWorks Inc., Natick, Massachusetts, USA). A single linear regression was performed to model the relationship of the stimuli lighting (predictor variable) with the (baseline-relative) change in TDT_20_ (outcome variable). The startle responses of 72 larval zebrafish were assessed and quantified in 10 runs of the experimental paradigm using incrementing kLux levels (0.5, 1, 2, 4, 6, 8, 10, 12, 14, 16), against an age-matched control group (N = 72) that was not stimulated with additional (baseline-relative) light (Supplementary Information).

## Supplementary Information

Supplementary Information.
