# Supplementary material for: Light-stimulus intensity modulates startle reflex habituation in larval zebrafish
Source: Sci Rep. 2021 Nov 17;11:22410. doi: 10.1038/s41598-021-00535-9 (PMC8599482; doi:10.1038/s41598-021-00535-9)
Supplement: Supplementary file 1 — Supplementary Information. [file 41598_2021_535_MOESM1_ESM.pdf]

Run	Control			Stimulated		
	kLux	M	SE	kLux	M	SE
**1**	0	18.6152	1.4967	0.5	18.8579	1.6165
**2**	0	16.6668	1.3574	1	15.7123	1.2247
**3**	0	19.3005	1.6278	2	18.2873	1.4691
**4**	0	16.3719	1.365	4	19.512	1-5657
**5**	0	13.5732	1.2615	6	18.8759	1.5465
**6**	0	16.6505	1.2954	8	21.0003	1.5962
**7**	0	14.3241	1.2818	10	22.3410	1.655
**8**	0	13.797	1.3492	12	25.1261	1.6607
**9**	0	15.4862	1.1103	14	24.5054	1.8124
**10**	0	17.141	1.2418	16	27.6797	1.7715

Table 1. Descriptive statistics (mean and SE) for the mean TDT_{20} for each group, at each level of the within-subject factor (light stimulus intensity).
